# Prevalence of Impacted Teeth in Saudi Patients Attending Dental Clinics in the Eastern Province of Saudi Arabia: A Radiographic Retrospective Study

**DOI:** 10.1155/2020/8104904

**Published:** 2020-09-01

**Authors:** Abdulaziz Alamri, Nasser Alshahrani, Abdullah Al-Madani, Suliman Shahin, Muhammad Nazir

**Affiliations:** ^1^Department of Preventive Dental Sciences, College of Dentistry, Imam Abdulrahman Bin Faisal University, P.O. Box 1982, Dammam 31441, Saudi Arabia; ^2^Biomedical Dental Sciences Department, College of Dentistry, Imam Abdulrahman Bin Faisal University, P.O. Box 1982, Dammam 31441, Saudi Arabia; ^3^Dental Hospital, College of Dentistry, Imam Abdulrahman Bin Faisal University, P.O. Box 1982, Dammam 31441, Saudi Arabia

## Abstract

**Aim:**

To evaluate the prevalence of impacted teeth in Saudi patients and compare between male and female subjects.

**Method:**

This cross-sectional study comprised of Saudi patients who attended dental clinics in major hospitals in the Eastern Province of Saudi Arabia. Patients' dental records and panoramic radiographs were reviewed retrospectively. Impacted teeth excluding third molars and spaces occupied by primary, permanent, and transmigrated teeth were recorded from panoramic radiographs. The Pearson chi-squared test was performed to determine gender differences regarding impacted teeth and spaces occupied by other teeth.

**Results:**

The study included radiographs of 539 patients with a mean age of 23.3 ± 10.8 years. Seventy-one patients (13.2%) had at least one impacted tooth. The total number of impacted teeth was 115 in the sample, out of which 91 (79.1%) were in the upper arch and 24 (20.8%) in the lower arch. Fifty-eight maxillary canines (50.4%) were impacted making them the most commonly impacted teeth, followed by 21 upper second premolars (18.2%) and 14 lower second premolars (12.2%). More females (70.7%) than males (29.3%) had impacted teeth (*P*=0.82). Of 61 spaces occupied, 35 (57.4%) were occupied by permanent teeth, 24 (39.3%) by primary teeth, and 2 (3.3%) by transmigrated teeth. Greater proportions of spaces were occupied in female than male participants (*P* > 0.05).

**Conclusion:**

There was a high prevalence of impacted teeth in Saudi patients. The canines were the most commonly impacted teeth followed by the second premolars. Females demonstrated a higher occurrence of impacted teeth than males. Early detection of impacted teeth can help prevent malocclusion and maintain a healthy dentition.

## 1. Introduction

Tooth impaction is defined “as a condition in which a tooth is prevented from eruption by some physical barrier in the eruption path” [1]. It is a frequent phenomenon that has been widely reported in the literature [2–5]. However, there are variations in the prevalence of impacted teeth in different parts of the world and their distribution in upper and lower jaws [6–8]. It was also reported that the prevalence of impacted teeth was more frequent in females than males [7]. In addition, different factors are associated with the tooth impaction which includes different age groups, the timing of tooth eruption, ethnicity or regions of study participants, and radiographic evaluation criteria [6, 9].

It is important to understand the role of impactions of different teeth in the etiology of various types of malocclusions, which can affect the movement of teeth, functional occlusion, and esthetic smile [8, 10]. For example, the impaction of maxillary canine can increase the risk of root resorption of adjacent lateral incisors, gingival infections, and cystic follicular lesions [11]. It is known that the canine impaction is one of the most prevalent dental anomalies [6, 8, 10]. After impacted third molars, maxillary permanent canines are the most commonly impacted teeth [12]. The incidence of maxillary canine impaction is 20 times higher than mandibular canine impaction [13].

In Saudi Arabia, researchers investigated the occurrence of canine and third molar impactions in different regions of the country [14–19]. Afify and Zawawi evaluated the prevalence of dental anomalies including impacted teeth in patients in Jeddah, Saudi Arabia [8]. However, the literature is scant about the prevalence of impactions of different teeth in Saudi populations in the Eastern Province of Saudi Arabia. The aim of the study, therefore, was to examine the prevalence and patterns of tooth impaction in Saudi patients in the Eastern Province of Saudi Arabia. In addition, the distribution of tooth impactions was compared in male and female subjects.

## 2. Materials and Methods

Ethical approval of this retrospective study was obtained from the institutional review board of Imam Abdulrahman Bin Faisal University, Dammam. A sample of 578 patients was estimated to be adequate for the study. The sample size was estimated on the assumptions of 4% margin of error, 95% confidence level, 50% response distribution, population size (*N* ≈ 15,000), and 80% of the power of the study. The subjects were the patients who attended the dental clinics of the major hospitals in the Eastern Province of Saudi Arabia. The records of the patients were obtained from Armed Forces Hospital at King Abdulaziz Airbase, King Fahd Teaching Hospital, and College of Dentistry at Imam Abdulrahman Bin Faisal University, Dammam. Patients' dental records and radiographs were examined retrospectively in order to record the impaction of incisors, canines, premolars, and molars (except third molars). The demographic details such as patient's age, gender, and residential information, were obtained from dental records.

Panoramic radiographs were examined carefully by well-trained and experienced dentists. The calibration sessions were held for two dentists, and their recordings were compared with a senior faculty member in the College of Dentistry. The interexaminer reliability agreement test was performed prior to the study, and reproducibility was considered good (Kappa 0.85). In addition, an experienced radiologist supervised the evaluation of radiographs, which was performed using a transparency projector under constant lighting conditions or constant degree of contrast for digital radiographs without magnification. A case definition was used for impacted teeth. The impacted teeth were those teeth that were prevented from eruption within path of eruption due to a physical barrier such as the adjacent teeth, bone, or soft tissue [6, 7]. Teeth were considered impacted if they remained in the jaw for more than two years beyond their average eruption time [6].

The records were included in the analysis if patients had permanent dentition, and the roots of the impacted teeth were fully formed in the radiographs. The cases in the archived records that met the inclusion and exclusion criteria were included in the study. Saudi patients were included in the study. The patients were excluded from the study if they exhibited one or more pathological situations (endocrinal deficiency such as hypothyroidism, hypopituitarism, trauma, or fracture of the jaw) and hereditary diseases or syndromes such as Down's syndrome and cleidocranial dysostosis. These patients were excluded because the normal growth of permanent dentition can be affected due to these conditions. The impactions of primary teeth and third molars were excluded from the study. An evaluation of impactions of different teeth excluding third molars is important for orthodontic treatment planning.

Data were gathered and analyzed using the SPSS software (IBM SPSS Statistics for Windows, Version 22.0, Armonk, NY: IBM Corp). Incomplete or missing data were excluded from the statistical analysis. Descriptive statistics included frequencies, percentages, means, and standard deviations. A chi-squared test was performed to compare the proportions of impacted teeth and spaces occupied between male and female study participants. A *P* value <0.05 was considered statistically significant.

## 3. Results

The study analyzed data of 539 patients with a mean age of 23.3 ± 10.8 years. There were 158 (29.3%) male and 381 (70.7%) female patients in the study. A total of 71 patients had at least one impacted tooth, and the prevalence of impaction was 13.2% (Table 1).

The evaluation of radiographs showed that there were 115 teeth impacted among study participants, and 91 teeth (79.1%) were in the upper arch and 24 (20.8%) in the lower arch. Fifty-eight maxillary canines (50.4%) were impacted making them the most commonly impacted teeth, followed by 21 upper second premolars (18.2%) and 14 lower second premolars (12.2%). In the maxilla, canines were the most commonly impacted (58 teeth), followed by second premolars (21 teeth) and the second molars (7 teeth). Among mandibular teeth, second premolars were most commonly impacted teeth (14 teeth), followed by canines (9 teeth) and then lateral incisor (1 tooth). Upper/lower central incisors, lower first premolars, upper/lower first molars, and lower second molars were not impacted. The prevalence of canine impaction was 9.1% as 49 of 539 patients had at least one impacted canine in upper and lower arches. Overall, there were 67 impacted canines in 49 patients (Table 2).

**Figure 1:**
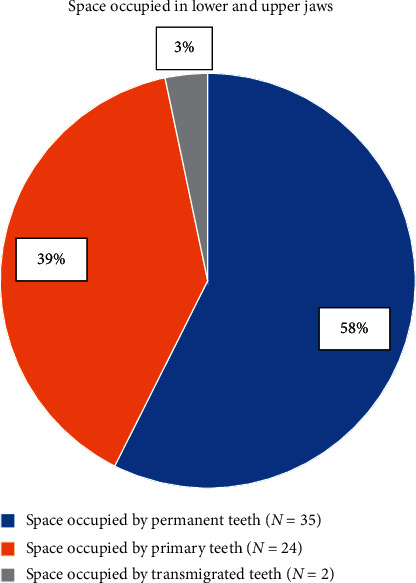
Distribution of spaces occupied by misplaced teeth in lower and upper jaws among study participants.

There were 61 spaces occupied by permanent, primary, and transmigrated teeth. Of these spaces, 35 (57.4%) were occupied by permanent teeth, 24 (39.3%) by primary teeth, and 2 (3.3%) by transmigrated teeth (Figure 1).

**Figure 2:**
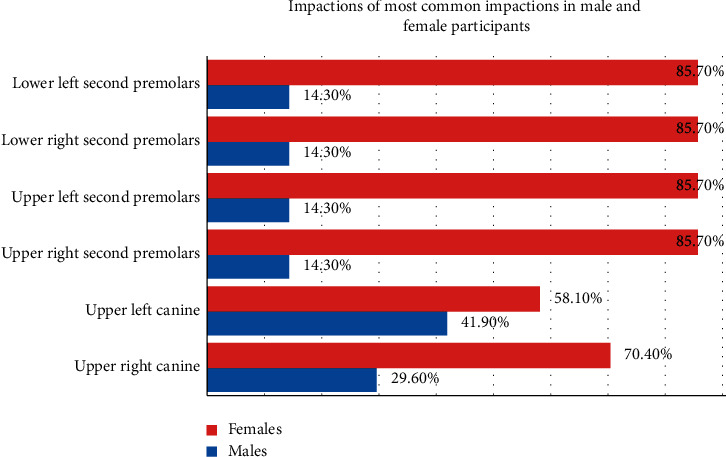
Gender distribution of most common impactions among study participants.

There were 20 (28.2%) males and 51 (71.8%) females with impacted teeth in the study with no statistically significant gender differences (*P*=0.82). In addition, three most common impactions were compared between male and female participants. The analysis showed there were 8 upper right canines in males compared with 19 in females, but these differences were not significant (*P*=0.97). Similarly, no statistically significant gender differences were observed with regards to other impacted teeth (Figure 2).

A lesser number of spaces were occupied by permanent and primary teeth in males than females; however, these differences were not significant (Table 3). The mean age of male participants (mean = 25.23, SD ± 13.01) was significantly higher than female participants (mean = 22.48, SD ± 9.7) (*P*=0.007).

## 4. Discussion

This retrospective analysis of radiographs showed that the prevalence of impacted teeth was 13.2% in Saudi patients in the Eastern Province of Saudi Arabia. The proportion of impacted teeth in our study was close to the prevalence estimates in other studies reported in the literature. For instance, Fardi et al. reported that 13.7% of impacted teeth was observed among the Greek population [6]. Aitasalo et al. evaluated patients' data in the University of Turku in Finland and stated that 14.1% of the studied population had impacted teeth [2]. In the northern part of India, Patil and Maheshwari reported that 16.8% of subjects were diagnosed with impacted teeth [10]. On the other hand, a recent similar study showed that 44.1% of cases had at least one impacted tooth in the central part of Iran [20]. Similarly, 28.3% of 7486 patient radiographic records revealed impacted teeth in Chinese in Hong Kong [7]. The prevalence of impacted teeth was 21.1% in a retrospective investigation of 878 digital orthopantomograms of subjects from Jeddah, Saudi Arabia [8]. However, the prevalence of impacted teeth was 2.5% in patients attending the College of Dentistry, Taibah University, Madinah, Saudi Arabia [21]. These variations in the prevalence of impacted teeth in different studies can be attributed to the diagnostic criteria used to define impaction and recruitment of study participants including different age groups and sample sizes.

From the orthodontic point of view, the impaction of canine is important for the prevention of malocclusion and maintenance of esthetics. In Saudi Arabia, the impaction of canines was studied by many researchers. In 1993, AlZahrani studied a sample of 4,898 Saudi patients and reported that 175 patients (3.6%) had at least one impacted canine [22]. Afify and Zawawi examined patient records in Jeddah and found 3.3% of patients with impacted canines [8]. In 2014, Mustafa reported that the prevalence of canine impaction was 1.44% in adult patients who visited the College of Dentistry, King Khalid University, Abha [23]. Another study from Saudi Arabia by Alrwuili included patients attending an orthodontic center in Al-Jouf and showed that 97 of 2239 subjects had impacted canines (4.33%), which were most commonly located in the maxilla [18]. In 2017, a retrospective analysis of panoramic radiographs of patients by Melha et al. showed that 3.65% of patients had canine impaction [24]. Alhammadi et al. (2018) reported canine impaction in 1.9% of patients attending the College of Dentistry, Jazan University, Saudi Arabia [25]. In Najran, Saudi Arabia, Alyami et al. observed canine impaction in 5.35% of patients [26]. In Turkey, 3.58% of patients were diagnosed with canine impaction after a review of 4500 consecutive panoramic radiographs [27]. Yemitan conducted a study on patients visiting the orthodontic clinic of a university teaching hospital in Nigeria and found that 45 of 460 subjects (9.8%) had at least one canine impacted [28].

Likewise, canines were the most common impacted teeth (9.1%) followed by premolars in the present study. In accordance with the results of our study, the impaction of canine was the most common (9.7%) followed by premolars in India [10]. Similarly, impacted canines were the most prevalent impacted teeth followed by premolars in Greeks [6]. The Chinese populations also demonstrated similar patterns of canine and premolar impactions [7].

In this study, impacted teeth were diagnosed in 20 male and 51 female subjects. However, our analysis showed no statistically significant differences in the occurrence of impacted teeth between male and female study participants, which agrees with the findings of other similar studies [6, 7, 9, 29]. Fardi et al. detected the existence of more impacted teeth in females (54.1%) than males (45.9%), but there were no significant gender differences [6]. Chu et al. demonstrated impacted teeth with a male to female ratio of 1 : 1.2 [7]. Among Brazilian patients, Pedro et al. observed no significant association between gender and impaction of teeth; however, authors identified strong influences of age and type of tooth on tooth impaction [9]. Recently, Arabion et al. also found no significant difference in the prevalence of impacted teeth between male (42.6%) and female (57.4%) patients in Iran [20].

High prevalence estimates of premature loss of primary teeth were reported in different parts of the world. Premature loss of primary teeth was 51% in Saudi Arabia [27], 40.54% in Yemen [28], 34.46% in India [29], and 24.7% in Brazil [30]. Premature loss of primary molars is a common phenomenon. Primary molars have increased susceptibility to *S. mutans* colonization due to early eruption and anatomical features, which predispose them to a carious attack. This may result in early loss of primary molars if left untreated [30]. When there is early loss of primary teeth, then adjacent teeth move in the extracted space. This leads to loss of space and reduction in arch length, which hinders the normal eruption of permanent teeth, thus causing their impactions. Crowding of teeth and asymmetry of dental arch are also sequelae of primary teeth loss [31]. Premature loss of primary teeth is the most common cause of impactions in the present study. Most spaces were closed due to permanent teeth (57.4%) in our study.

To our knowledge, this study is the first to provide valuable information about the prevalence of different types of impactions except for third molars in Saudi patients. The study filled knowledge gap on the distribution of impacted teeth in the Eastern Province of Saudi Arabia. In particular, the impactions of canines, premolars, and molars were highlighted in the study. However, there were some limitations to the study as well. Females tend to seek for more dental care than males because they are more concerned with their esthetics. This might have led to an increased number of radiographic records of females included in our study and the resultant higher impaction rate among females than males. Although, our sample size was close to some other previous studies [16, 28]. However, it was possible that a larger sample size in our study could affect the prevalence of impacted teeth. The present study showed that one quarter (31.9%) of the patients was between the age of 20 and 30 years. This reflects that a considerable proportion of this young population was dentally aware and availed free dental services in the Eastern Province of Saudi Arabia. The inclusion of this large group of participants, however, could influence the prevalence figures in our study. It is known that a random sample is appropriate to represent the population of the province and to provide accurate prevalence estimates of impacted teeth. However, a representative sample of the population was a challenge because exposing the randomly selected study participants to radiation is unethical and costly. Therefore, caution should be exercised when generalizing the study findings.

## 5. Conclusions

The study found high prevalence of impacted teeth in Saudi patients attending dental clinics in the Eastern Province of Saudi Arabia. Impactions occurred more frequently in the upper than the lower arch. The canines were the most commonly impacted teeth followed by second premolars. The most common cause of impaction was the premature loss of primary teeth. Females demonstrated greater impactions than males. Early detection of impacted teeth should be performed to prevent malocclusion and to maintain a healthy and normal dentition, which would improve esthetics and masticatory functions.

## Figures and Tables

**Table 1:** Descriptive statistics of the study participants.

Factors	*N* (%)
	(*N* = 539)
Gender	
Male	158 (29.3)
Female	381 (70.7)
Impaction	
Yes	71 (13.2)
No	468 (86.8)
	Mean ± SD
Age	23.29 ± 10.8

**Table 2:** Impaction of different teeth among study participants.

Impaction of upper teeth	*N* (%)	Impaction of lower teeth	*N* (%)
	Impacted teeth in both arches (*N*) = 115		Impacted teeth in both arches (*N*) = 115
Central incisor	0	Central incisor	0
Right		Right	
Left		Left	
Lateral central incisor	3 (2.6)	Lateral central incisor	1 (0.8)
Right	1	Right	0
Left	2	Left	1
Canines	58 (50.4)	Canines	9 (7.8)
Right	27	Right	5
Left	31	Left	4
First premolar	2 (1.7)	First premolar	0
Right	1	Right	
Left	1	Left	
Second premolar	21 (18.2)	Second premolar	14 (12.2)
Right	14	Right	7
Left	7	Left	7
First molar	0	First molar	0
Right		Right	
Left		Left	
Second molar	7 (6.1)	Second molar	0
Right	4	Right	
Left	3	Left	
Total number of impacted teeth in upper arch	91	Total number of impacted teeth in lower arch	24

**Table 3:** Gender distribution of spaces occupied among study participants.

Space occupied	Males	Females	*P* value
Space occupied by permanent teeth	6 (17.1)	29 (82.9)	0.102
Space occupied by primary teeth	9 (37.5)	15 (62.5)	0.367
Space occupied by transmigrated teeth	1 (50)	1 (50)	0.520

## Data Availability

The SPSS data file of this study is available from the corresponding author upon request.
